# Supplementary figures and images for: The effects of Cstb duplication on APP/amyloid-β pathology and cathepsin B activity in a mouse model
Source: PLoS One. 2021 Jul 22;16(7):e0242236. doi: 10.1371/journal.pone.0242236 (PMC8297773; doi:10.1371/journal.pone.0242236)

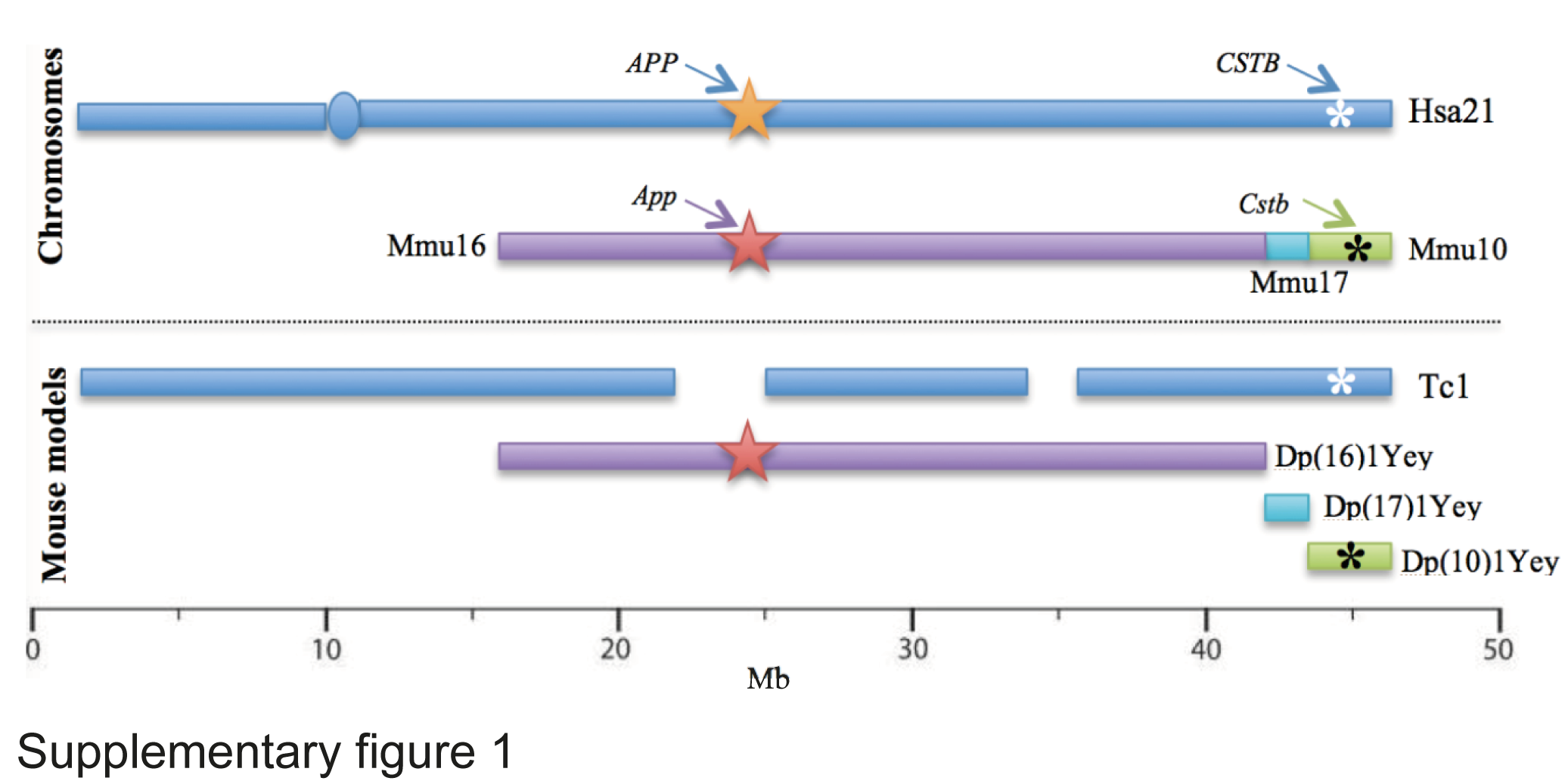

Supplement: S1 Fig — The regions of Mmu16, 17, and 10 are aligned with their corresponding regions on the long arm of Hsa21, along a megabase pair (Mb) scale. The Tc1 mouse model is represented with breakpoints excluding the Hsa21 genes that are not functionally expressed. The approximate position of the human and mouse APP and CSTB genes are indicated with arrows. (TIF) [file pone.0242236.s001.tif]
